# Aberrant regulation of autophagy disturbs fibrotic liver regeneration after partial hepatectomy

**DOI:** 10.3389/fcell.2022.1030338

**Published:** 2022-10-26

**Authors:** Yuan-E. Lian, Yan-Nan Bai, Jian-Lin Lai, Ai-Min Huang

**Affiliations:** ^1^ Department of Pathology and Institute of Oncology, The School of Basic Medical Sciences, Fujian Medical University, Fuzhou, China; ^2^ Department of Pathology, The Affiliated Union Hospital of Fujian Medical University, Fuzhou, China; ^3^ Shengli Clinical Medical College of Fujian Medical University, Department of Hepatobiliary and Pancreatic Surgery, Fujian Provincial Hospital, Fuzhou, China

**Keywords:** autophagy, liver regeneration, fibrotic liver, partial hepatectomy, verapamil

## Abstract

Reports indicate that autophagy is essential for maintaining hepatocyte proliferative capacity during liver regeneration. However, the role of autophagy in fibrotic liver regeneration is incompletely elucidated. We investigated the deregulation of autophagic activities in liver regeneration after partial hepatectomy using a CCl4-induced fibrosis mouse model. The baseline autophagic activity was significantly increased in the fibrotic liver. After 50% partial hepatectomy (PHx), liver regeneration was remarkably decreased, accompanied by increased hepatocyte size and binuclearity ratio. Moreover, the expression of autophagy-related proteins was functionally deregulated and resulted in a reduction in the number of autophagosome and autophagosome–lysosome fusions. We further showed upregulation of autophagy activities through verapamil administration, improved hepatocyte proliferation capacity, and restricted cellular hypertrophy and binuclearity ratio. In conclusion, we demonstrated that the impairment of liver regeneration is associated with aberrant autophagy in fibrotic liver and that enhancing autophagy with verapamil may partially restore the impaired liver regeneration following PHx.

## Introduction

The understanding of liver regeneration has considerably improved over the past decades. Liver regeneration is characterized by an extraordinary and well-orchestrated regenerative process following partial hepatectomy (PHx) in a normal liver (Fausto et al., 2012; Tanaka and Miyajima, 2016; Michalopoulos and Bhushan, 2021). Liver fibrosis is the most common pathological alteration when the liver is insulted by numerous etiologies (e.g., drugs, alcohol, and viruses). The regeneration capacity of the fibrotic liver sharply declines yet the underlying mechanisms remain poorly understood (Andıran et al., 2000; Mann et al., 2001; Kele et al., 2013). Either in experimental or clinical studies, the poor outcome is generally attributed to compromised hepatocyte proliferation (Nagasue et al., 1987; Andiran et al., 2000; Kuramitsu et al., 2013; Aierken et al., 2020). As the fibrotic liver has a reduced regeneration capacity, the extent of the hepatectomy is limited in terms of its clinical application due to the potential risk of acute liver failure and death.

Autophagy, an evolutionarily well-conserved catabolic pathway in processing self-degradation of intracellular material, physiologically maintains cellular homeostasis and remodeling. Autophagy acts ubiquitously at a basal level and can be induced in response to intra- or extra-cellular stimuli (Klionsky et al., 2021). Pathologically, the autophagic activity is remarkably upregulated in patients with liver cirrhosis or in experimental models with liver fibrosis (Hernandez-Gea et al., 2012; Hung et al., 2015). The autophagic activity may be induced for profibrogenesis in the liver. In zebrafish caudal fin regeneration, the autophagic activity significantly increases and plays an indispensable role in cell fate determination and proliferation (Varga et al., 2014). In the liver, the autophagic regulation of regeneration is important for maintaining metabolic balance and pre-venting hepatocyte senescence (Toshima et al., 2014). A plethora of evidence indicates that the depletion of the protein products of some critical autophagy-related genes, including Atg5 (Toshima et al., 2014), Atg7 (Lin et al., 2015; Romermann et al., 2020), and cathepsin L (Sato et al., 2019), leads to impairment of hepatocyte mitosis and proliferation. By contrast, liver regeneration can be improved through stimulation of autophagic promoters (Lin et al., 2015; Romermann et al., 2020; Lai et al., 2021).

It is paradoxical that the upregulation of the autophagic activity in liver regeneration and liver fibrosis is uninformative to interpret the impaired regeneration capacity in the fibrotic liver. Although the molecular mechanisms of autophagy in regulating the liver regeneration process were described based on the normal liver, an investigation of autophagic regulation in fibrotic liver regeneration is still lacking. In this study, we developed a murine model of liver regeneration with mild or moderate degree of liver fibrosis. We observed that liver regeneration gradually declined with the increasing severity of fibrosis. In moderate liver fibrosis after 50% PHx, hepatocyte proliferation sharply decreased, accompanied with the apparent enlargement of hepatocytes, which was a sign of hepatocyte hypertrophy (Miyaoka et al., 2012). Unexpectedly, the expression of autophagy-related proteins in the moderate fibrotic liver significantly differed from that of the liver under normal or mild fibrotic contexts. These results indicate that aberrant autophagic activities may have a poor impact on the process of liver regeneration. We also investigated whether the corrected autophagic activity by verapamil administration can partially restore the regeneration capacity.

## Materials and methods

### Construction of carbon tetrachloride-induced fibrotic mice

All the mice used in this study were of C57BL/6 background and were raised at the Animal Experimental Center of Fujian Medical University under previously described conditions (Lai et al., 2021).

Carbon tetrachloride (CCl4) liver fibrosis was induced in male C57BL/6 mice weighing 21.3 ± 0.2 g using previously described CCl4 mixture regimen (intraperitoneal injection with 20% CCl4 at 100 or 150 mg dosage twice a week for seven consecutive weeks). Mild and moderate liver fibrosis were set according to the proposed fibrosis staging criteria (Zhao et al., 2008). Mice with moderate fibrosis were further intraperitoneally injected with verapamil (5 mg per kg bodyweight for 10 days) prior to 50% PHx for the induction of autophagy (Lai et al., 2021).

### Partial hepatectomy

PHx was performed in mice 1 week after CCL4 injection. Mice were anesthetized with 2% isoflurane continuous inhalation and subjected to approximately 50% PHx by removing the left lateral lobe, superior and inferior right lobes, or 70% PHx by removing the left lateral and median lobes, after midventral laparotomy (Martins et al., 2008). The residual liver was obtained from defined time points after PHx: 0, 6, 12, 24, 36, 48, and 72 h.

### Western blot analysis

Frozen liver tissues were homogenized, and the total protein extracts were prepared according. SDS-PAGE was performed using standard western blotting protocols. The antibodies used in this study are listed in Supplementary Table S1. The proteins were then scanned using an enhanced chemiluminescence detection system (Thermo Fisher Scientific Inc., Waltham, MA, United States), and the relative density of immunoreactive bands was quantitated using ImageJ (National Institutes of Health, Bethesda, MA, United States).

### Histology and immunofluorescence staining

Formalin-fixed liver tissue was processed and stained with hematoxylin and eosin or Masson’s trichrome stain for the denotation of liver fibrosis.

Paraffin-fixed sections were subjected to immunofluorescence staining using standard protocols. The primary antibodies used in this study are listed in Supplementary Table S1. Nulcei were stained with Hoechst 33342 (CST, Beverly, MA, United States). Images were obtained using a microscope (ApoTome.2, Zeiss, Jena, Germany) and the accompanying software, Axiovision (version 4.7.2).

### Measurement of cell size and binuclearity ratio

With the combination of nuclei staining and cellular outline staining by Hoechst 33342 and phalloidin-labeled actin, respectively, hepatocytes can be recognized at the threshold of nuclear circularity (≥0.7) by the sectional area of 100–1500 µm^2^. The cellular size was calculated in 300–400 individual hepatocytes per mouse using Image J (Miyaoka et al., 2012; Bou-Nader et al., 2020). To determine the number of nuclei, mononuclear and binuclear hepatocytes were quantified on 10 random high-power fields (about 3000 cells in total) on scans of stained sections. The pathologists who performed these calculations were blinded to the treatment group.

### Transmission electron microscopy

The methods used for transmission electron microscopy (TEM) were described previously (Lai et al., 2021).

### Statistical analysis

All statistical analyses were performed using the “survival” and “ggplot2” packages in R (version 3.6.1). All variables are expressed as the mean ± standard deviation. Continuous variables were compared using the nonparametric Wilcoxon test for independent samples or the parametric paired t-test for paired samples. Survival was calculated using the Kaplan–Meier method, and between-group differences in survival were compared using the log-rank test. Values of *p* < 0.05 were considered to indicate significance.

## Results

### Increased autophagic activity in the murine fibrotic liver

**FIGURE 1:**
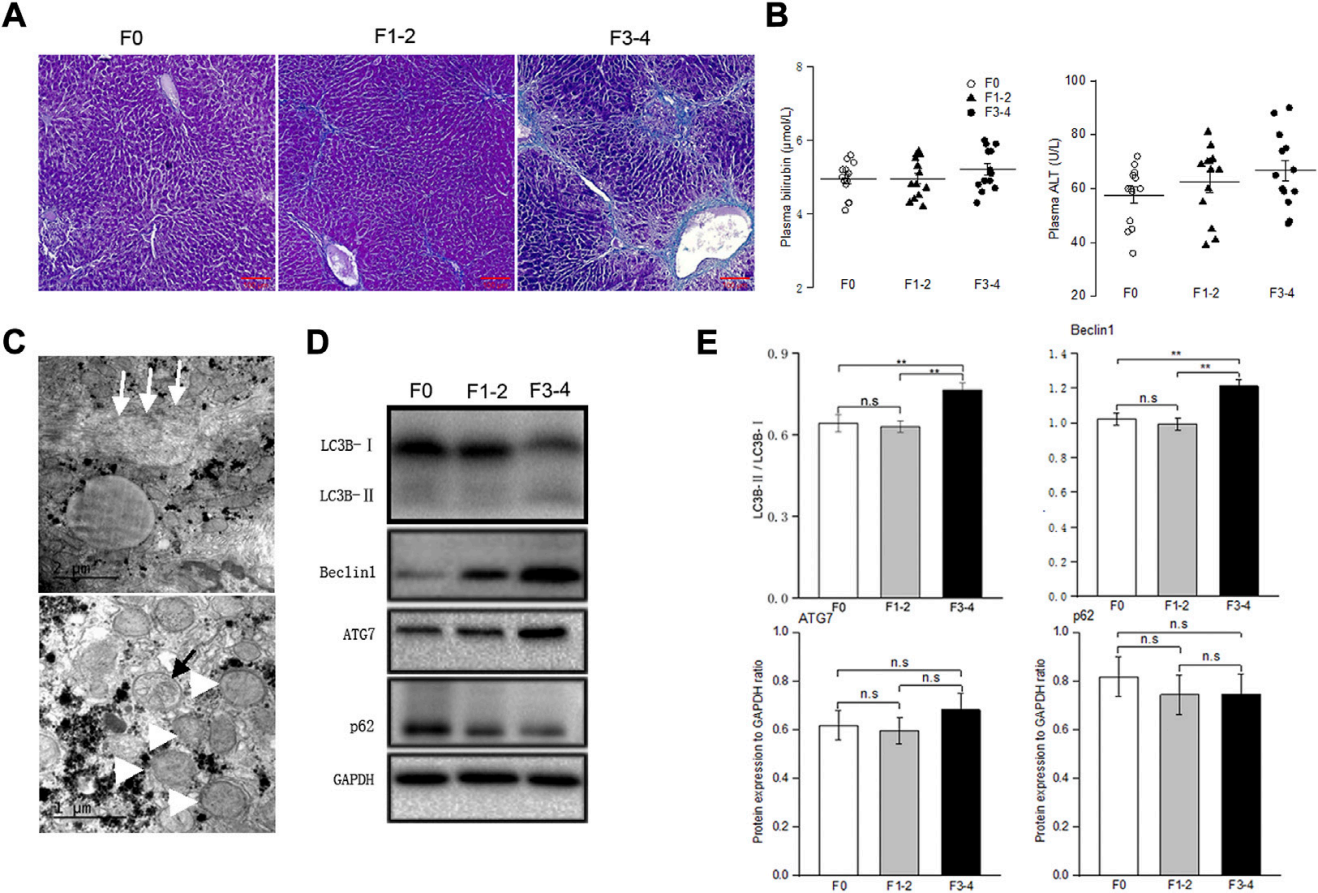
Baseline autophagic activities in fibrosis liver. **(A)**, Representative pictures of Masson trichrome staining; scale bar, 100 μm. **(B)**, Plasma bilirubin and alanine transaminase levels were unremarkably changed between groups; n = 10–12. **(C)**, Transmission electron microscopic images of autophagosomes (black arrow) and enlarged mitochondria (white arrowhead) in the fibrotic liver, collagenous fiber (white arrow) may be seen at subcellular level. **(D)**, Representative Western blot analysis depicts fibrosis and autophagic markers, including LC3B, Beclin1, ATG7, p62 (GAPDH used as loading control); GAPDH, glyceraldehyde-3-phosphate dehydrogenase. **(E)**, The intensity of protein bands was quantitated using the ImageJ software version 1.53c (NIH, Bethesda, MD; http://imagej.nih.gov/ij), and normalization of LC3B-II to LC3B-I, and Beclin1, ATG7 and p62 to GAPDH was shown. n.s, non-significance; **p < 0.01.

To determine the basal autophagy of the fibrotic liver, we generated experimental mice in F1–2 or F3–4 stages of liver fibrosis (Figures 1A,C). The severity of the liver fibrosis was assessed through fibrosis staging criteria that specifically apply to murine CCl4-induced fibrosis models (Zhao et al., 2008). Since there is no universal acceptance of fibrosis staging between species and etiology, liver fibrosis in F1–2 stage is basically equivalent to mild fibrosis, which is characterized by short fibrous tissue in the central venule (C) or C-C fibrotic septa appearance in histopathology. The F3–4 stage is equivalent to moderate fibrosis, which is characterized by multiple C-C fibrotic septa incompletely or completely dividing hepatic lobules into pseudo lobules. The concentration of plasma bilirubin and alanine transaminase in mild or moderate fibrotic mice increased rather unremarkably (Figure 1B). TEM showed markedly increased number of autophagosomes in fibrotic livers and enlarged mitochondria, compared with those in nonfibrotic livers (F0 group) (Figure 1C). To confirm that the increased autophagosome formation reflects activated autophagy in fibrotic liver tissue, we determined the protein expression beclin 1 through western blotting. As an initial recruiter protein for nucleation of autophagosomes, beclin 1 expression in the F3–4 group was significantly higher than that in the F0 or F1–2 groups (Figures 1D,E). LC3B protein is another autophagosome-related marker, and the conversion of the cytoplasmic isoform LC3B-I to the membrane-associated isoform LC3B-II reflects the induction of the autophagic flux. The abundance of the LC3B-I protein gradually decreased as the liver fibrosis progressed, while the converted LC3B-II/I ratio in F3–4 liver fibrosis significantly increased (Figures 1D,E). The expression of Atg7 and p62 was determined and showed an unremarkable difference between nonfibrotic and fibrotic livers. Collectively, these observations indicated a greater autophagic process in the progression of liver fibrosis.

### Impaired regeneration capacity in the murine fibrotic liver

**FIGURE 2:**
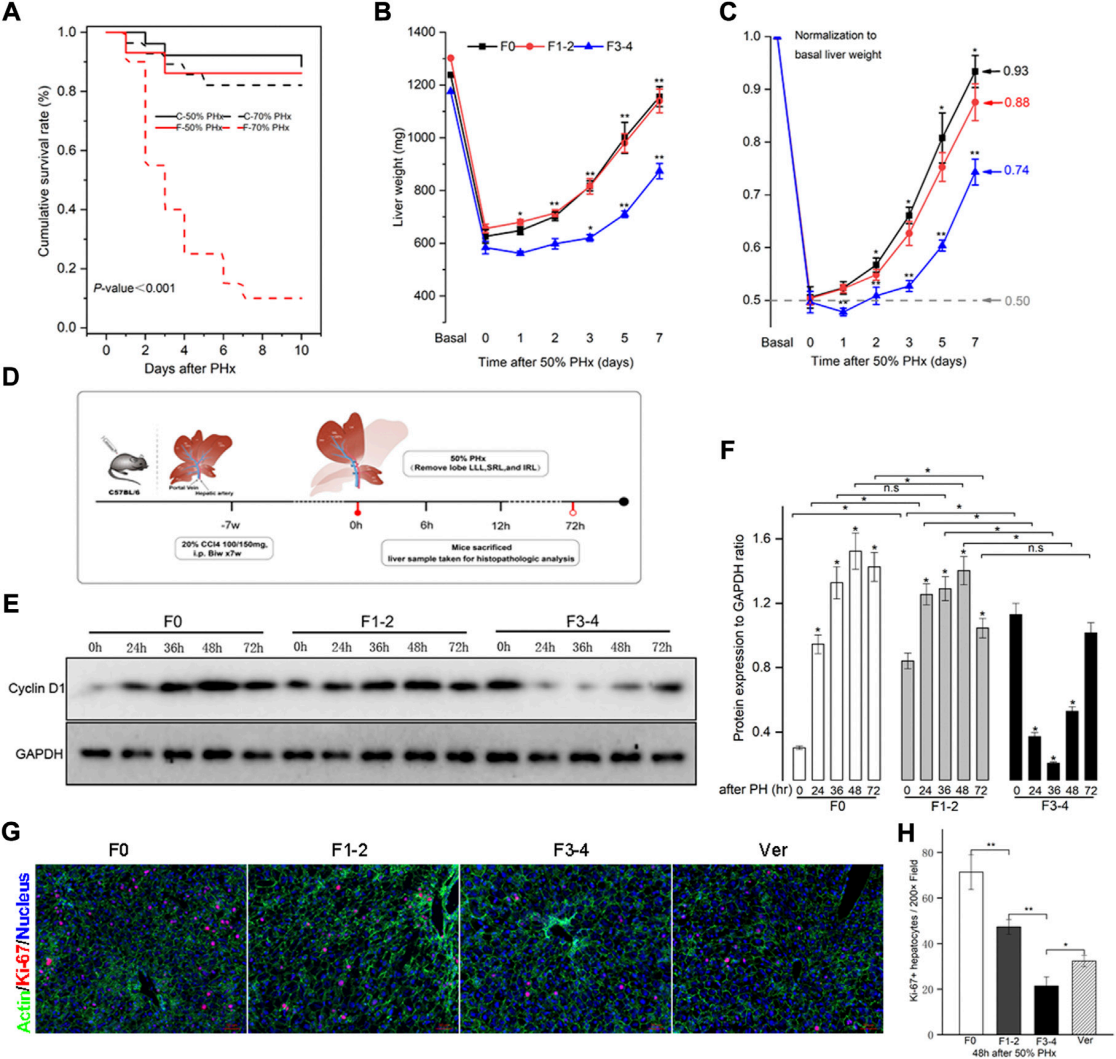
Liver fibrosis compromised survival and impaired liver regeneration capacity after PHx. CCl4-induced C57BL/6 fibrotic mouse and their counterpart littermates underwent PHx. **(A)**, Survival rate was comparable except in F3-4 fibrotic mouse underwent 70% PHx. Survival rate was calculated using the Kaplan-Meier method and was 71.8% in total mice (74/103). C, control mice with nonfibrotic liver; F, experimental mice with F3-4 fibrotic liver. **(B)**, Experimental scheme. F1-2 or F3-4 liver fibrosis was induced by chronic CCl4 injections for 7 weeks with twice dosage of 100 mg or 150 mg in C57BL/6 males, respectively. One week after last CCl4 injection, 50% PHx was performed by removing love LLL, SRL and IRL; the liver samples were harvested at the determined time points. LLL, the left lateral lobe; SRL, the superior right lobe; IRL, the inferior inferior right lobe. **(C)**, Liver weight of F0, F1-2 and F3-4 mice after 50% PHx at the determined time points. **(D)**, After normalization to basal liver weight, impaired recovery of liver mass was shown regarding to fibrotic stage. **(E)**, Hepatic cyclin D1 expression after 50% PHx was detected by western blotting. GAPDH, glyceraldehyde-3-phosphate dehydrogenase. **(F)**, The results by densitometry for cyclin D1 expression was plotted. **(G)**, Representative immunofluorescent images from liver sections stained with proliferation marker Ki-67 showed significantly low replicative activity in F1-2 or F3-4 fibrotic versus F0 livers at 48 hours after 50% PHx, while the proliferative activity was partially restored in F3-4 fibrotic mice with verapamil administration. Ver, verapamil; Scale bar, 50 μm **(H)**, Quantification of Ki-67 positive hepatocytes was plotted. Ten randomly HPFs were assessed in sections from five individual mice. HPF, high-power field. h(r), hour; n.s, non-significance; *p < 0.05; **p < 0.01.

PHx at 70% is the classic stimulating method to induce intense hepatocyte proliferation during normal liver regeneration. This type of major liver volume loss, however, caused unfavorably high mortality in fibrotic mice (Figure 2A). PHx at 50%, triggered liver regeneration less intensely as previously reported and made the postoperative vitality of the fibrotic mice comparable to PHx at 70% in normal littermates (Bonninghoff et al., 2012). Therefore, mice with 50% PHx were eventually selected secondary to the construction of CCl4-induced fibrosis (Figure 2B). As shown in Figure 2C, the usual liver parenchyma in mice with 50% PHx experienced a rapid regeneration process and restored nearly the initial liver weight at postoperative day 7 (Supplementary Table S2). Interestingly, the liver regenerative response and volume restoration of the F1–2 group were comparable to those of the F0 group but, significantly decreased in the F3–4 group. Normalization of the regeneration to basal liver mass was further processed to eliminate the bias from variant liver weight of nonfibrotic and fibrotic mice. As normalized to 0.50 owing to 50% PHx at the 0-day-time point, the regeneration index increased significantly at an early regenerative stage and regained 0.93 and 0.88 of the initial weight 7 days after PHx in F0 and F1–2 groups, respectively (Figure 2C). However, the index curve in the F3–4 group was less steep and only regained 0.74 at the corresponding time points (Figure 2D). These observations indicated impaired regenerative potential as a result of liver fibrosis.

The expression of the cyclin D1 protein is essential for cell cycle transition in the early stages of liver regeneration. As previously reported (Michalopoulos and Bhushan, 2021), cyclin D1 was induced and peaked at 48 h after PHx in F0 as well as in F1–2 groups (Figures 2E,F). In comparison, although the basal level of cyclin D1 in the F3–4 group was higher than that in the other groups, its expression declined during the early stages of liver regeneration, and the peak was delayed at 72 h after PHx. To further check hepatocyte proliferation, the immunofluorescent staining of Ki-67 at 48 h after PHx was performed. Quantification of labeled hepatocytes showed markedly decreased proliferation in the F3–4 group (Figures 2G,H). These observations indicated that the regenerative capacity was impaired and even deteriorated as liver fibrosis progressed.

### Hepatocyte enlargement and increased binuclearity ratio number during liver regeneration

**FIGURE 3:**
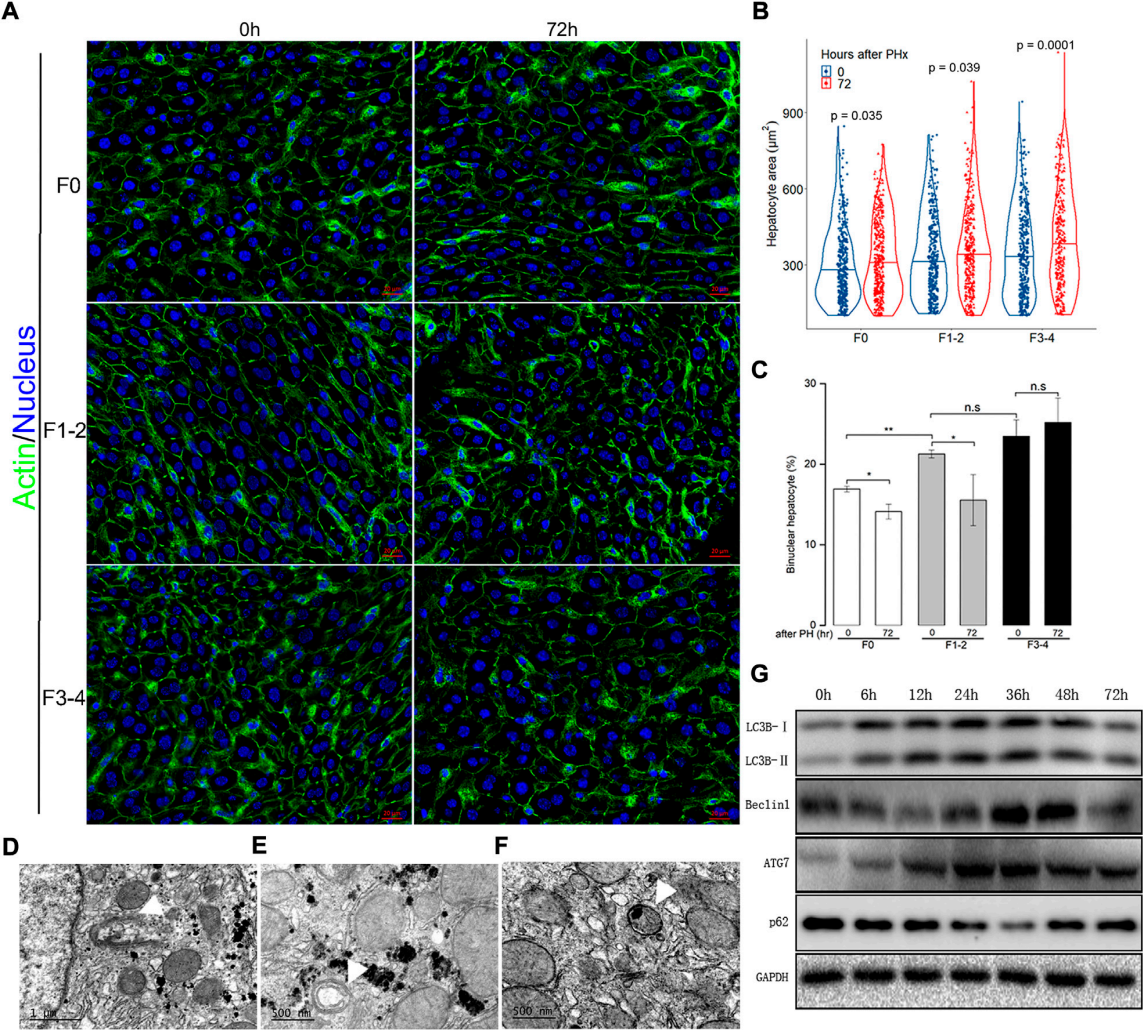
Analysis of hepatocyte size and cellular ploidy in F0, F1-2 and F3-4 fibrotic mice 72 hours after 50% PHx, and autophagic activity in the early phase of normal liver regeneration. **(A)**, Representative images of hepatocytes after 50% PHx. Staining of actin (green) and nuclei (blue) of hepatocytes were stained by Hoechst 33342 and Phalloidin respectively. Scale bar, 20 μm. **(B)**, Violin plot of hepatocyte size showed significant cell hypertrophy at 72 hours after 50% PHx, compared to baseline time point. The cellular size was calculated in 300–400 individual hepatocytes per mouse using ImageJ software. **(C)**, The number of binuclear hepatocyte was calculated and the plotted histograms showed insignificantly changed ratio of binuclear hepatocytes in F3-4 fibrotic livers. The mononuclear and binuclear hepatocytes were quantified on 10 random HPFs (about 3000 cells) on scans of stained sections. HPF, high-power field. h(r), hour; n.s, non-significance; *p < 0.05; **p < 0.01. D-F, Electron-microscopic feature of autophagosome formation (**(D)**, scale bar, 1μm), maturation (**(E)**, scale bar, 500 nm) and fusion with lysosome (**(F)**, scale bar, 500 nm) during F0 fibrotic regeneration at 48 hours after 50% PHx (white arrowhead). **(G)**, Expression of the autophagic proteins was demonstrated in F0 mice at 0-72 hours after 50% PHx.

Besides hepatocyte proliferation and division—a compensatory response of liver hypertrophy through hepatocyte size enlargement, especially at an early stage after PHx—is of equal importance during liver regeneration (Miyaoka et al., 2012; Romermann et al., 2020). With an appropriate threshold of circularity of nuclei and actin staining, the area of each hepatocyte can be calculated precisely. As expected, hepatocytes became significantly enlarged 72 h after 50% PHx during normal liver regeneration compared to that at the baseline time point (Figures 3A,B and Supplementary Table S3). Hepatocyte enlargement in the F1–2 group was comparable (both *p* = 0.04) but was more evident in the F3–4 group (*p* = 0.0001). During homeostasis, adult hepatocytes maintained a specific and stable proportion of binuclearity; however, during the regeneration process, this proportion markedly declined. Our observation fits well with these reports in that the number of binuclear hepatocytes reduced at 72 h after 50% PHx (Figures 3A,C) (Li et al., 2009; Miyaoka et al., 2012). However, both the cellular size and binuclearity ratio increased in fibrotic liver as a result of CCl4 insult. Interestingly, the number of nuclei in the cell decreased in the F1–2 group but increased in the F3–4 group during liver regeneration. These observations indicated that fibrosis markedly impeded the regeneration process and might contribute to the failure of hepatocyte proliferation in the advanced stage.

### Aberrant autophagic activity might be functional during fibrotic liver regeneration

Previous evidence indicated a dual regulatory role of autophagy in liver regeneration and fibrogenesis (Toshima et al., 2014; Hung et al., 2015). Since aberrant autophagy induced liver hypertrophy and compromised hepatocyte proliferation, there seemed to be a link between regulatory autophagy and hepatocyte enlargement and number of nuclei. The autophagic activity during the regenerative process, including electron microscopic features of autophagosome formation, maturation, fusion with lysosome, and upregulated expression of critical autophagy-related proteins, are presented in Figures 3D–G.

**FIGURE 4:**
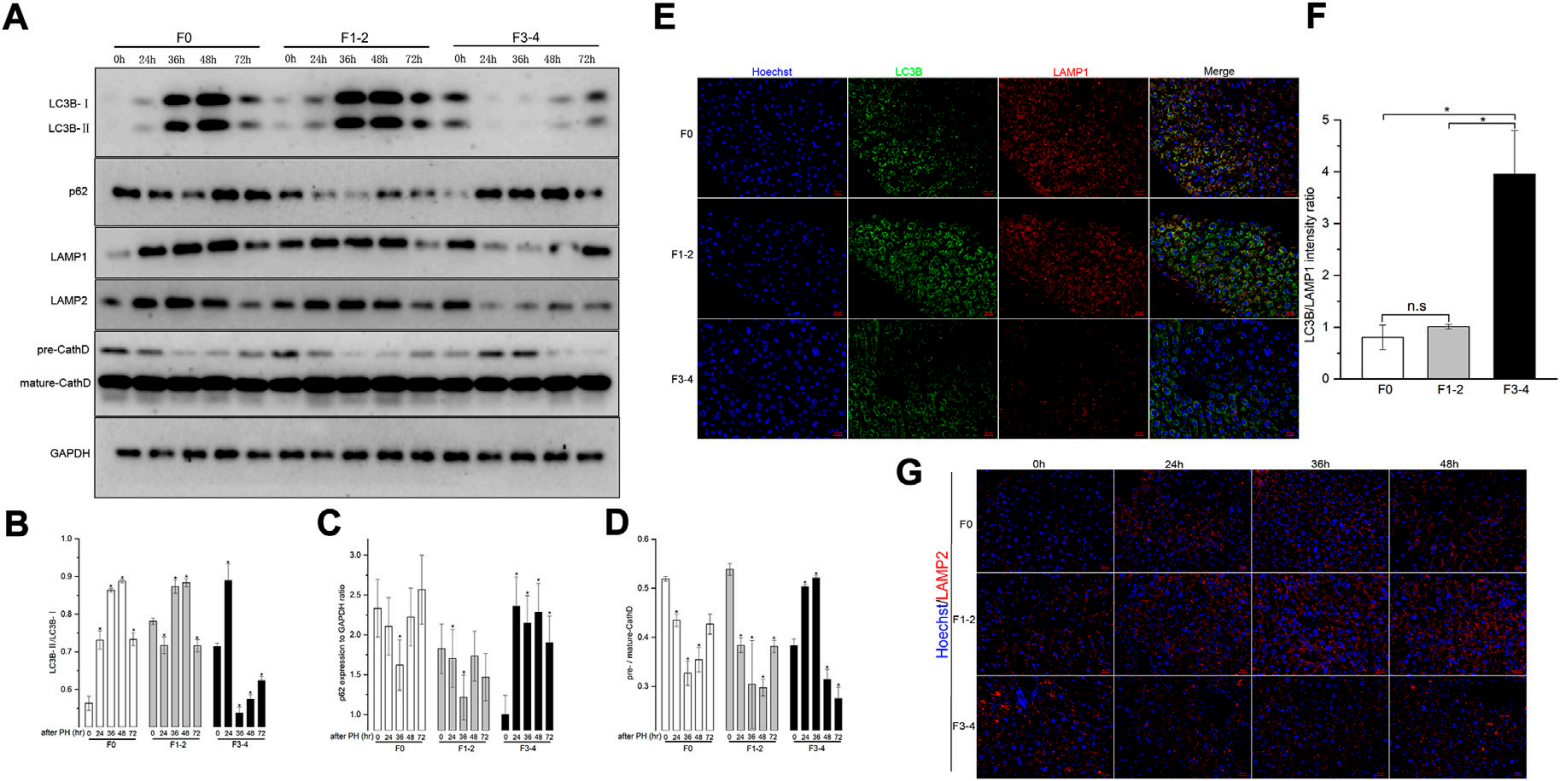
Analysis of autophagy levels in fibrotic and nonfibrotic livers. **(A)**, Representative Western blot analysis depicts that expression peak of autophagic proteins (LC3B, p62, LAMP1, LAMP2, Cathepsin D) was basically delayed as fibrosis progressed. **(B–D)**, The results by densitometry for LC3B-II/LC3B-I **(B)**, p62 **(C)**, and pre-/mature-Cathepsin D **(D)** was plotted respectively. The comparison was performed in each post-PHx time point versus baseline 0 hour. CathD, Cathepsin D; GAPDH, glyceraldehyde-3-phosphate dehydrogenase. **(E)**, Dual-immunofluorescence staining of LC3B (green) and LAMP-1 (red) in F0, F1-2 and F3-4 fibrotic livers. Nuclei (blue) of hepatocytes was stained by Hoechst 33342. Scale bar, 20 μm. **(F)**, The plotted Histograms for LC3B/LAMP1 immunofluorescent intensity ratio indicated relatively small number of autophagosome-lysosome fusions in F3-4 fibrotic livers. **(G)**, Immunofluorescence staining of LAMP2 (red) and nuclei (blue) in F0, F1-2 and F3-4 fibrotic livers. Nuclei (blue) of hepatocytes was stained by Hoechst 33342. Scale bar, 20 μm; n.s, non-significance; *p< 0.05.

**FIGURE 5:**
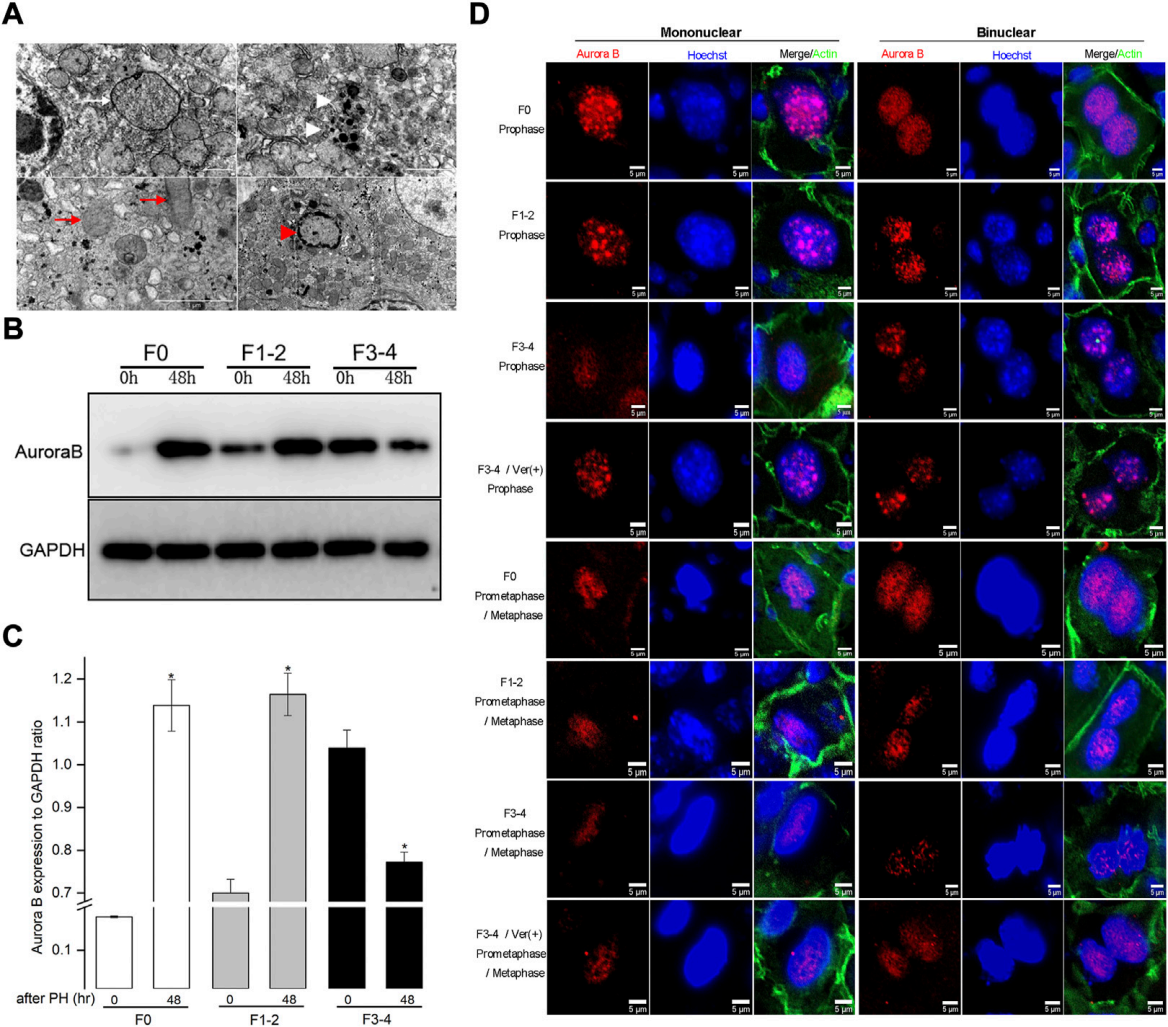
Impaired autophagic activities in F3-4 fibrotic liver regeneration and its association with mitosis. **(A)**, Representative features of impaired autophagic activities by electron-microscopically, formation of autophagosome (white arrow), autolysosome (white arrowhead), flatten mitochondria (red arrows) and apoptotic body (red arrowhead). **(B)**, Representative Western blot analysis depicts the expression of mitotic Aurora B protein in F0, F1-2, and F3-4 fibrotic mice. **(C),** The results by densitometry for Aurora B expression was plotted respectively. The comparison was performed at 48 hours after 50% PHx versus baseline 0 hour. GAPDH, glyceraldehyde-3-phosphate dehydrogenase; *p < 0.05. **(D)**, Immunofluorescent staining of Aurora B (red) in mononuclear (left panel) and binuclear hepatocytes (right panel) in F0, F1-2, and F3-4 fibrotic livers. Representative images at prophase (upper four rows) and prometaphase/metaphase (lower four rows) of mitosis were shown. The images of 4th and 8th row represented Aurora B staining after verapamil administration in F3-4 fibrotic mice. Nuclei (blue) of hepatocytes was stained by Hoechst 33342. Ver, verapamil; Scale bar, 5 μm.

Differences in autophagic activities between the normal and fibrotic regeneration processes were further determined. In F0 mice, the conversion of cytoplasmic LC3B-I to autophagosome-bound LC3B-II and the expression of p62 protein peaked at 48 h after 50% PHx, and indicated upregulation of the autophagic flux during the regeneration process (Figures 4A–D). A similar pattern of LAMP-2 and cathepsin D expression indicated an increase in lysosome numbers and activated lysosomal function. To assess the autophagosome–lysosome fusion step, subcellular localization of the lysosomal marker LAMP-1 and the autophagosomal marker LC3B was performed at the same time points using dual immunofluorescence labeling. The immunofluorescent colocalization of punctate LC3B and LAMP-1 staining with an intensity ratio of 0.80 indicated an appropriate fusion into autolysosome at the final stage of autophagy (Figures 4E,F). In the F1–2 group, the corresponding autophagy expression during liver regeneration showed a comparable tendency to that of the F0 group. In the F3–4 group, the expression pattern showed evident differences, including delayed peak of LC3B and other proteins at 72 h after 50% PHx; this indicated delayed formation of the autophagosome and reduced immunofluorescent colocalization intensity of LC3B and LAMP-1 staining, which indicated relatively small number of autophagosome–lysosome fusions (Figure 4). Through TEM, although the formation of autophagosomes and autolysosomes reflected an autophagic process during F3–4 fibrotic liver regeneration, accompanied events of apoptotic bodies and increased flattening of mitochondria implied the presence of impaired autophagy (Figure 5A). These observations indicated that aberrant autophagic signals in the moderate fibrotic liver might account for hepatocyte enlargement and increased binuclearity ration.

### Inhibition of mitotic responses during fibrotic liver regeneration

Autophagy has been found to be involved in the mitotic response of hepatocytes during the regeneration process (Toshima et al., 2014). To better understand the hepatocyte enlargement caused by aberrant autophagy, we focused on mitosis in hepatocytes during liver regeneration through examining the expression of Aurora B, which plays a centrally kinase-activated role in mitotic progression and cell division (Ruchaud et al., 2007). At 48 h after 50% PHx, the expression of Aurora B protein significantly increased in F0 and F1–2 groups, compared with its level before hepatectomy (Figures 5B,C). Conversely, the expression of Aurora B in the F3–4 group significantly decreased as expected. Regarding the critical time of the maximal entry into mitosis during liver regeneration, the intracellular distribution of Aurora B and morphology of nuclei can be used to represent each mitotic stage (Ruchaud et al., 2007; Miyaoka et al., 2012; Miyaoka and Miyajima, 2013). We investigated the expression of Aurora B in hepatocytes 48 h after 50% PHx using immunofluorescence (Figure 5D). In F0 pro- and prometaphase hepatocytes, the punctuated distribution of Aurora B at the chromosomes was clearly visible, for both mononuclear and binuclear hepatocytes. Aurora B remained well detectable with a slight decline in F1–2 mitotic hepatocytes. Interestingly, as liver fibrosis progressed, an obvious decline in the signals for Aurora B in F3–4 mitotic hepatocytes was observed. These observations indicated that Aurora B may be functionally affected, resulting in enlarged volume and declined proliferation of hepatocytes in fibrotic background due to aberrant autophagy.

### Impaired liver regeneration could be partially restored through increased autophagy

**FIGURE 6:**
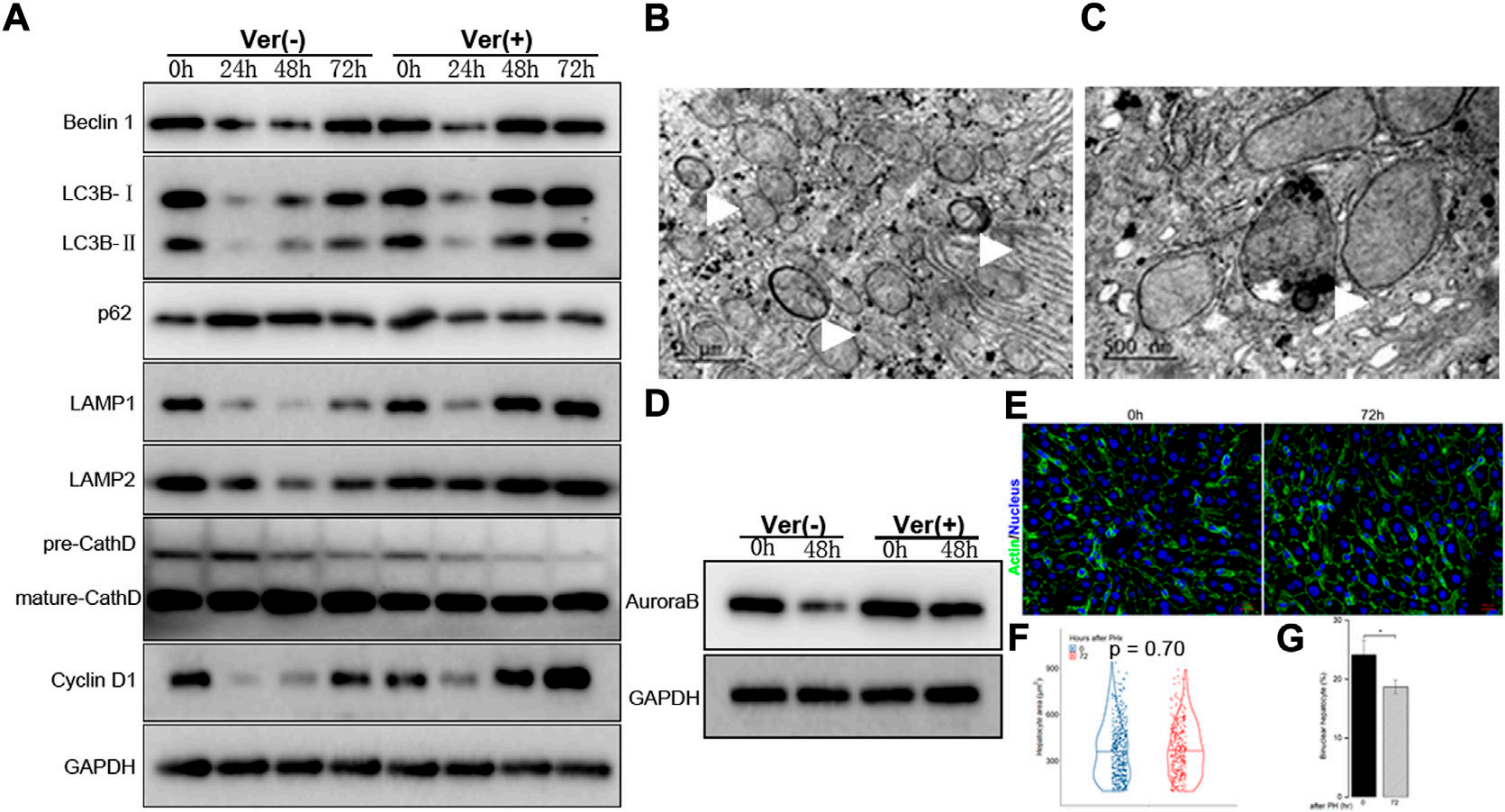
Liver regeneration capacity was partially restored under corrected autophagy after 50% PHx. **(A)**, Representative Western blot analysis depicts that expression of autophagic proteins (Beclin1, LC3B, p62, LAMP1, LAMP2, Cathepsin D) and cyclin D1 in the F3-4 fibrotic liver of mice with or without verapamil administration. **(B–C)**, Representative features of upregulated autophagy by electron-microscopically, including increased formation of autophagosome (**(B)**, white arrows), autolysosome (**(C)**, white arrow). **(D)**, Representative Western blot analysis depicts that expression of Aurora B. **(E)**, Representative images of hepatocytes in the F3-4 fibrotic liver of mice with or without verapamil administration. Staining of actin (green) and nuclei (blue) of hepatocytes were stained by Hoechst 33342 and Phalloidin respectively. Scale bar, 20 μm. **(F)**, Violin plot of hepatocyte size showed insignificant cell hypertrophy at 72 hours after 50% PHx, compared to baseline time point. **(G)**, The number of binuclear hepatocyte was calculated and the plotted histograms showed a significantly declined ratio of binuclear hepatocytes after verapamil administration. Ver, verapamil; GAPDH, glyceraldehyde-3-phosphate dehydrogenase; h, hour; *p < 0.05.

Based on the findings presented above, we hypothesized that autophagy induction might be beneficial in improving the impaired regenerative capacity of hepatocytes in mice with moderate fibrosis. Thus, verapamil, which has been shown to pharmacologically restore the autophagosome–lysosome fusion among other benefits, was administered prior to 50% PHx in F3–4 mice (Park et al., 2014; Lai et al., 2021). Remarkably, the verapamil treatment induced Atg-related protein expression earlier than that in the non-treated mice; the peak of beclin 1, LC3B, and LAMP-1/2 expression occurred 48 h after 50% PHx (Figure 6A). Through electron microscopy, the number of autophagosomes and autolysosomes were observed to increase, but there were virtually no diseased mitochondria or apoptotic bodies (Figures 6B,C and data not shown). These observations indicated that verapamil administration improved the aberrant autophagy activity in the fibrotic liver.

Accordingly, we explored whether the corrected autophagy would improve regenerative capacity. No mortality was caused by the verapamil treatment, and the expression of cyclin D1 protein increased from 48 to 72 h after 50% PHx (Figure 6A). In verapamil-treated mice, the Ki-67 labeling index was significantly higher than that in non-treated mice (Figures 2G,H). Similarly, the expression of Aurora B (Figure 6D) and immunofluorescent punctuated Aurora B signals (Figure 5D) were partially recovered, for both mononuclear and binuclear pro-/prometaphase hepatocytes. Based on micro-morphology, the hepatocyte size in verapamil-treated mice was insignificantly enlarged, and hepatocytes exhibited a descending binuclearity ratio postoperatively (Figures 6F,G and Supplementary Table S3) compared to those of non-treated mice. These observations indicated that hepatocyte proliferation was partially restored as a result of autophagy enhancement by verapamil during liver regeneration.

## Discussion

In this study, we investigate the relationship between autophagy and liver regeneration in 50% PHx models with different degrees of fibrosis. To the best of our knowledge, we found, for the first time, that fibrotic mice have reduced autophagic activity in the early stages of liver regeneration (Figure 4). The suppression of autophagy is intensified when liver fibrosis progresses. In fact, the tendency of autophagic activity in mice with mild fibrosis was generally consistent with that in normal mice, which is upregulated in the early stages of liver regeneration and is critical for the protection of hepatocyte proliferation and organ homeostasis (Toshima et al., 2014; Sato et al., 2019; Romermann et al., 2020). By contrast, downregulated autophagy was observed in mice with moderate fibrosis with hepatocyte enlargement and increased binuclearity ratio, which indicated impaired regeneration capacity (Figure 3). Surprisingly, the impaired regeneration could be partially restored through pharmacological autophagic induction (Figure 6). In mice with moderate liver fibrosis, we found that corrected autophagy causes Aurora B expression of mononuclear or binuclear pro-/prometaphase hepatocytes to increase, which mechanistically indicated the entry into mitosis (Figures 5, 6). Furthermore, corrected autophagy increased the expression of proliferation-related proteins (cyclin D1 and Ki-67) and improved hepatocyte enlargement and binuclearity ratio (Figures 2, 6). Thus, aberrant autophagy in mice with liver fibrosis impaired hepatocyte proliferation during liver regeneration and could be partially ameliorated through pharmacological autophagic induction.

Liver fibrosis is an adaptive response attributed to chronic hepatic insults with drugs, alcohol, and viruses. Unlike normal liver regeneration, the pathophysiology of fibrotic liver regeneration, characterized by comprehensive failure of hepatocytes to replicate, remained to be elucidated. In agreement with previous reports (Hernandez-Gea et al., 2012; Kuramitsu et al., 2013; Hung et al., 2015), the baseline upregulation of autophagy in CCl4-induced liver fibrosis and impairment of liver regeneration after 50% PHx in the fibrotic mouse was indicated (Figures 1. In that context, alteration of autophagy seems to play a “double-edged sword” role in the pathogenesis of liver diseases. In patients with α1-antitrypsin deficiency, autophagy is specifically triggered for the intracellular degradation of the misfolded AT proteins to prevent cellular enlargement and hepatomegaly (Marciniak and Lomas, 2010). The activation of autophagy is similarly triggered by insults with alcohol (Gual et al., 2017), CCl4 (Hernandez-Gea et al., 2012), and steatosis (Singh et al., 2009; Czaja, 2011), whereas the hepatic depletion of autophagy causes metabolic and energetic dysfunction of hepatocytes, which can lead to hepatomegaly, fibrosis, or carcinogenesis. In another study, the activation of autophagy was identified as a profibrogenic factor in activated hepatic stellate cells (Hung et al., 2015). Accordingly, autophagy may be a protective or non-protective mechanism depending on the functional effects of each liver cell type (Hung et al., 2020). Thus, as the predominant parenchymal cell, previous studies indicated that hepatocytes play a central role in liver regeneration through upregulated autophagy (Toshima et al., 2014; Lin et al., 2015; Sato et al., 2019; Romermann et al., 2020). The reversal of the autophagy tendency was immensely different from the normal progression after hepatectomy in our F3–4 fibrotic mice, indicating suppression of autophagic signals in the hepatocyte in the fibrotic microenvironment during liver regeneration.

The deletion of autophagy-related genes prominently decreases hepatocyte proliferation (Toshima et al., 2014; Sato et al., 2019; Romermann et al., 2020). Our results showing decreased cyclin D1 and Ki-67 expression together with impaired autophagy were similar to previous observations during regeneration in the moderate fibrotic mice (Figures 2E–G). Furthermore, hepatocyte size enlargement (hypertrophy) and increased number of binuclear hepatocytes were observed (Figures 3A,B). Unconventionally, hypertrophy was an alternative regeneration pathway such as hepatocyte proliferation in liver regeneration (Miyaoka et al., 2012). It was claimed that size enlargement of hepatocytes is the first process by which liver regenerates, especially in PHx with less resected liver volume, hypertrophy of hepatocytes plays critical, even solely role on liver regenerative process before cell division. The enlargement of hepatocytes could be attributed to dysregulation of autophagy (Toshima et al., 2014; Romermann et al., 2020). In normal liver regeneration, cell size grew larger as regeneration proceeding. Hepatocytes are prevented from becoming senescent and intracellular organelles are functional well under upregulated autophagy in the early phase of liver regeneration. Rationally, hypertrophy is a compensatory response as physiological levels of autophagy are required for normal cell size. However, in L-Atg5 KO mice, this responsive hypertrophy was more severe which might suggest problematic degradation of cumulatively dysfunctional organelles in the status of defective autophagy (Toshima et al., 2014). This phenomenon was indicated in another Atg7-deficient PHx mice model (Romermann et al., 2020), as well as by patients with α1-antitrypsin deficiency for the autophagic inability of degradation of the misfolded AT proteins (Marciniak and Lomas, 2010). In our study, obvious enlargement of hepatocytes and downregulated autophagy coexisted in F3-4 fibrotic mice. It is interesting to note that enlargement of hepatocytes can be partly reversed by enhanced autophagy with verapamil administration. Liver regeneration is improved through upregulated autophagy which might hint reversely problematic disposal of damaged intracellular organelles; thus, cell size was normally enlarged rather than excessively hypertrophic (Toshima et al., 2014; Sato et al., 2019; Romermann et al., 2020). Unlike physiological status, insufficient or excessive levels of autophagy would lead to mismatch in cell enlargement (Vellai et al., 2008; Neufeld, 2012).

The profile for hepatocyte binuclearity ratio reflects its proliferation and differentiation status in various physiological process (Toyoda et al., 2005). In livers from adults, approximately 20% of hepatocytes have double nuclei (Li et al., 2009). The binuclearity ratio remains stable in dormant mature hepatocytes but declines in normal regenerative process apparently owing to replicative response from binuclear hepatocytes to mononuclear daughter cells (Gerlyng et al., 1993; Li et al., 2009). Our results in F0 regenerative liver are consistent with those of previous reports (Figures 3A–C; and Supplementary Table S3). Unlike in F0 regenerative liver, the ratio of binuclear hepatocytes increased instead in F3–4 regenerative liver at 72 h after 50% PHx. In this regard, the hepatocyte entering into the cell cycle does not necessarily indicate cell division; insufficient cytokinesis would prevent the conversion of polyploidization and cause a decrease in the number of binuclear hepatocytes (Guidotti et al., 2003; Miyaoka et al., 2012). The proliferation status of hepatocytes can be further revealed by investigating the expression of Aurora B between mitotic phases. According to the intracellular distribution of Aurora B and morphology of nuclei of hepatocytes, the punctuated distribution of Aurora B remarkably declined in F3–4 mitotic hepatocytes for both mononuclear and binuclear hepatocytes (Figure 5D).

As downregulated autophagy is associated with declined proliferation of hepatocytes during fibrotic liver regeneration, pharmacological restoration of autophagic signals was induced through verapamil administration (Park et al., 2014; Lai et al., 2021). The corrected autophagy included earlier expression of Atg-related proteins at 48 h after 50% PHx, increased number of autophagosomes and autolysosomes, and decreased numbers of diseased mitochondria or apoptotic bodies (Figures 6A–C). As a result, proliferative indices improved, especially the reduced number and degree of hypertrophic and polyploid hepatocytes (Figures 6D–G). Hepatocyte enlargement and descreasing number of binuclear hepatocyte suggested that hypertrophy together with proliferation jointly contribute to liver regeneration (Minamishima et al., 2002; Haga et al., 2005; Miyaoka et al., 2012; Miyaoka and Miyajima, 2013).

Two main factors challenge the experimental results and their robustness. The resection volume of the liver is the first affected factor. When 40%–70% of the liver is removed, the hepatocyte proliferation response is linearly correlated with the extent of volume loss (Bonninghoff et al., 2012). Thus, mice with 70% PHx constitute the classic murine model because this procedure produces a strong regenerative response postoperatively. Liver fibrosis is another factor; accumulating evidence indicates the impaired regeneration capacity of hepatocytes in fibrotic liver (Cao et al., 2009; Bonninghoff et al., 2012). Under preestablished experimental liver fibrosis, primary replication of hepatocytes is substantially impaired, and alternative regenerative pathways of intrahepatic or extrahepatic stem cells would be activated in mice with excessive liver volume loss (Kuramitsu et al., 2013). Nonetheless, most of the mice died postoperatively (Figure 2). Since the aim of our study was to explore the potential regenerative mechanism of mature hepatocytes under fibrotic conditions, limited hepatectomy (50%) triggered regeneration response although less intensely, and the postoperative vitality was maintained stably, which would have avoided positive selection of experimental animals. Hence, a more clinically relevant condition, such as 50% PHx, was eventually chosen as an experimental model.

In conclusion, we have demonstrated that the aberrant regulation of autophagy disturbs fibrotic liver regeneration after 50% PHx. Hepatocyte proliferation improved with verapamil by pharmacologically modulating autophagy, thereby reducing the number and degree of hypertrophic and polyploid hepatocytes.

## Data Availability

The raw data supporting the conclusions of this article will be made available by the authors, without undue reservation.
