# Supplementary material for: Aberrant regulation of autophagy disturbs fibrotic liver regeneration after partial hepatectomy
Source: Front Cell Dev Biol. 2022 Oct 26;10:1030338. doi: 10.3389/fcell.2022.1030338 (PMC9644332; doi:10.3389/fcell.2022.1030338)
Supplement: Supplementary file 1 [file Table2.docx]

Supplement S2: Liver mass recovery in F0, F1-2, and F3-4 fibrotic mice after 50% PHx (mg)

| Time point (days) | | | | Normalization to basal status | | |
| --- | --- | --- | --- | --- | --- | --- |
|  | F0 | F1-2 | F3-4 | F0 | F1-2 | F3-4 |
| Basal status | 1238±44 | 1302±40 | 1176±23 | 1 | 1 | 1 |
| 0 | 626±25 | 656±17 | 584±24 | 0.51±0.02 | 0.50±0.01 | 0.50±0.02 |
| 1 | 648±15 | 680±10^*^ | 562±8.4 | 0.52±0.01^*^ | 0.52±0.008 | 0.47±0.01^**^ |
| 2 | 702±16^**^ | 714±13^**^ | 598±19 | 0.57±0.01^*^ | 0.55±0.01^*^ | 0.51±0.02^**^ |
| 3 | 818±19^**^ | 816±30^**^ | 620±12^*^ | 0.66±0.02^*^ | 0.63±0.02^*^ | 0.52±0.01^**^ |
| 5 | 1000±59^**^ | 980±35^**^ | 710±12^**^ | 0.81±0.05^*^ | 0.75±0.03^*^ | 0.60±0.01^**^ |
| 7 | 1156±38^**^ | 1140±45^**^ | 840±29^**^ | 0.93±0.03^*^ | 0.87±0.03^*^ | 0.74±0.02^**^ |

Compared with time point 0 hour. *P < 0.05; **P < 0.01.
