# Supplementary material for: Aberrant regulation of autophagy disturbs fibrotic liver regeneration after partial hepatectomy
Source: Front Cell Dev Biol. 2022 Oct 26;10:1030338. doi: 10.3389/fcell.2022.1030338 (PMC9644332; doi:10.3389/fcell.2022.1030338)
Supplement: Supplementary file 2 [file Table3.docx]

Supplement S3: Hepatocyte area and Binuclear hepatocyte rate at 0 hour and 72 hours after 50% PHx in F0, F1-2, F3-4 and Ver_F3-4 fibrotic mice.

|  | Hepatocyte area (μm²) | | | Binuclear hepatocyte (%) | | |
| --- | --- | --- | --- | --- | --- | --- |
|  | 0H | 72H | P value | 0H | 72H | P value |
| F0 | 301.46±145.84 | 324.13±153.53 | 0.035 | 16.90±0.35 | 14.12±0.92 | 0.011 |
| F1-2 | 330.77±158.69 | 356.17±170.41 | 0.039 | 21.27±0.49 | 15.54±3.18 | 0.043 |
| F3-4 | 340.65±169.57 | 394.24±189.79 | 0.0001 | 23.47±2.01 | 25.19±3.04 | 0.23 |
| Ver_F3-4 | 367.96±182.97 | 373.02±170.52 | 0.70 | 24.14±2.44 | 18.68±1.17 | 0.021 |
